# What is the difference between the breakpoint graph and the de Bruijn graph?

**DOI:** 10.1186/1471-2164-15-S6-S6

**Published:** 2014-10-17

**Authors:** Yu Lin, Sergey Nurk, Pavel A Pevzner

**Affiliations:** 1 Department of Computer Science and Engineering, University of California, San Diego, 9500 Gilman Dr, CA 92093 La Jolla, USA; 2 Algorithmic Biology Laboratory, St. Petersburg Academic University, St. Petersburg, Russia; 3 St. Petersburg State University, St. Petersburg, Russia

**Keywords:** breakpoint graphs, de Bruijn graphs, genome rearrangements, genome assembly

## Abstract

The breakpoint graph and the de Bruijn graph are two key data structures in the studies of genome rearrangements and genome assembly. However, the classical breakpoint graphs are defined on two genomes (represented as sequences of synteny blocks), while the classical de Bruijn graphs are defined on a single genome (represented as DNA strings). Thus, the connection between these two graph models is not explicit. We generalize the notions of both the breakpoint graph and the de Bruijn graph, and make it transparent that the breakpoint graph and the de Bruijn graph are mathematically equivalent. The explicit description of the connection between these important data structures provides a bridge between two previously separated bioinformatics communities studying genome rearrangements and genome assembly.

## Introduction

The de Bruijn graph is a data structure first brought to bioinformatics as a method to assemble genomes from the experimental data generated by sequencing by hybridization [1]. It later became the key algorithmic technique in genome assembly [2,3] that resulted in dozens of software tools [4-12]. In addition, the de Bruijn graphs have been used for repeat classification [13], de novo protein sequencing [14], synteny block construction [15,16], multiple sequence alignment [17], and other applications in genomics and proteomics.

The breakpoint graph is a data structure introduced to study the reversal distance [18], which has formed the basis for much algorithmic research on rearrangements over the last two decades [19].

Since the connections between the breakpoint graphs and the de Bruijn graphs was never explicitly described, researchers studying genome rearrangements often do not realize that breakpoint graphs are merely de Bruijn graphs in disguise. As a result, they often do not know how to move from the traditional breakpoint graphs on *synteny blocks *to the breakpoint graphs on genomes (with "single nucleotide" resolution), particularly in the case of double-stranded *genomes *with inverted repeats. Likewise, researchers working in genome assembly are often unaware about the connections between the de Bruijn graphs and the breakpoint graphs. As a result, the exchange of ideas between these two communities has been limited. For example, Iqbal et al. [20] recently introduced the notion of the *colored de Bruijn graphs *that resulted in a popular Cortex assembler. While the notion of the colored de Bruijn graphs is essentially identical to the notion of the breakpoint graph, authors of [20] are probably unaware about this connection since they provided no references to previous genome rearrangement studies. This is unfortunate since various results about the breakpoint graphs (e.g., the connection between rearrangements and alternating cycles) remained beyond the scope of this very useful study.

Recently, genome rearrangement studies moved from the level of synteny blocks to the level of single nucleotides [21]. Likewise, genome assembly experts recently moved towards the analysis of structural variations and comparative assembly of related species based on the analysis of the de Bruijn graphs [20]. We thus argue that the time has come to explain that the breakpoint graphs and the de Bruijn graphs are two identical data structures (if one ignores a cosmetic difference between them) as they both represent specific instances of a general notion of the *A-Bruijn graph *introduced in [13]. The A-Bruijn graphs are based on representing genomes as sets of labeled paths and further gluing identically labeled edges (breakpoint graphs) or vertices (de Bruijn graphs) in the resulting paths.

**Figure 1:**
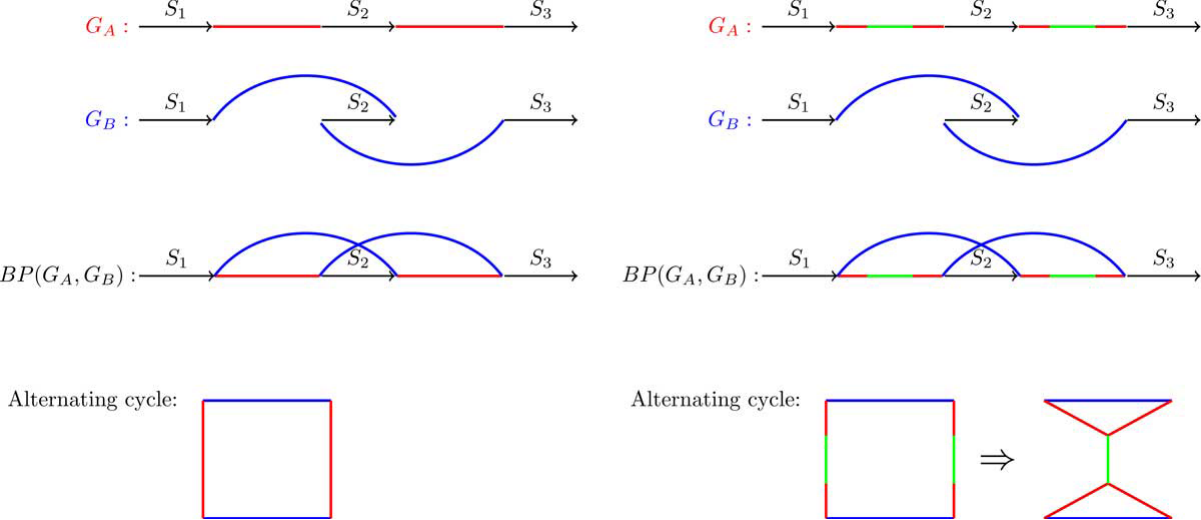
**Genomes *G_A _*= *S*_1_, *S*_2_, *S*_3 _and *G_B _*= *S*_1_, −*S*_2_, *S*_3 _(represented as bicolored paths) differ from each other by a single reversal of segment *S*_2_**. The breakpoint graph *BP*(*G_A_, G_B_*) and the alternating cycle constructed from genomes *G_A _*and *G_B _*(left: no repeats at the breakpoint regions; right, a pair of repeats colored in green at the breakpoint regions).

We argue that a unified framework covering both breakpoint and de Bruijn graphs is important to bridge the divide between researchers working with breakpoint graphs (that usually focus in rearrangements and ignore repeats) and researchers working with de Bruijn graphs (that usually focus on repeats and ignore rearrangements). In reality, there exists a complex interplay between rearrangements and repeats, e.g., LINE repeats and segmental duplications often trigger rearrangements [22-24]. However, this interplay is not explicitly revealed by the breakpoint graphs since they do not even encode repeats (repeats are intentionally masked at the synteny block construction step). For example, the interplay between LINEs and rearrangements cannot be derived from the breakpoint graph alone forcing Zhao and Bourque [23] to perform additional analysis. Our goal is to introduce the graphs that encode both rearrangements and repeats and immediately reveal this interplay. For example, encoding repeats present in the breakpoint regions (that may potentially trigger rearrangements) leads to gluing alternating cycles in the breakpoint graphs and requires development of new algorithms that integrate rearrangements and repeats. In such graphs, the classical *non-self-intersecting alternating cycles *formed by edges alternating between two colors (the workhorse of genome rearrangement studies) may turn into *self-intersecting *cycles formed by edges alternating between 3 colors, where the third color corresponds to repeated elements (see Figure 1). Nurk and Pevzner [25] recently used this framework to develop a new comparative genome analysis tool SPArcle and applied it to analyzing multiple bacterial strains resulting from the "controlled evolution" experiments [26]. SPArcle is based on SPAdes assembler and, in difference from Cortex, it uses ideas from the previous genome rearrangement studies (e.g., alternating cycles) to analyze the resulting A-Bruijn graphs.

**Figure 2:**
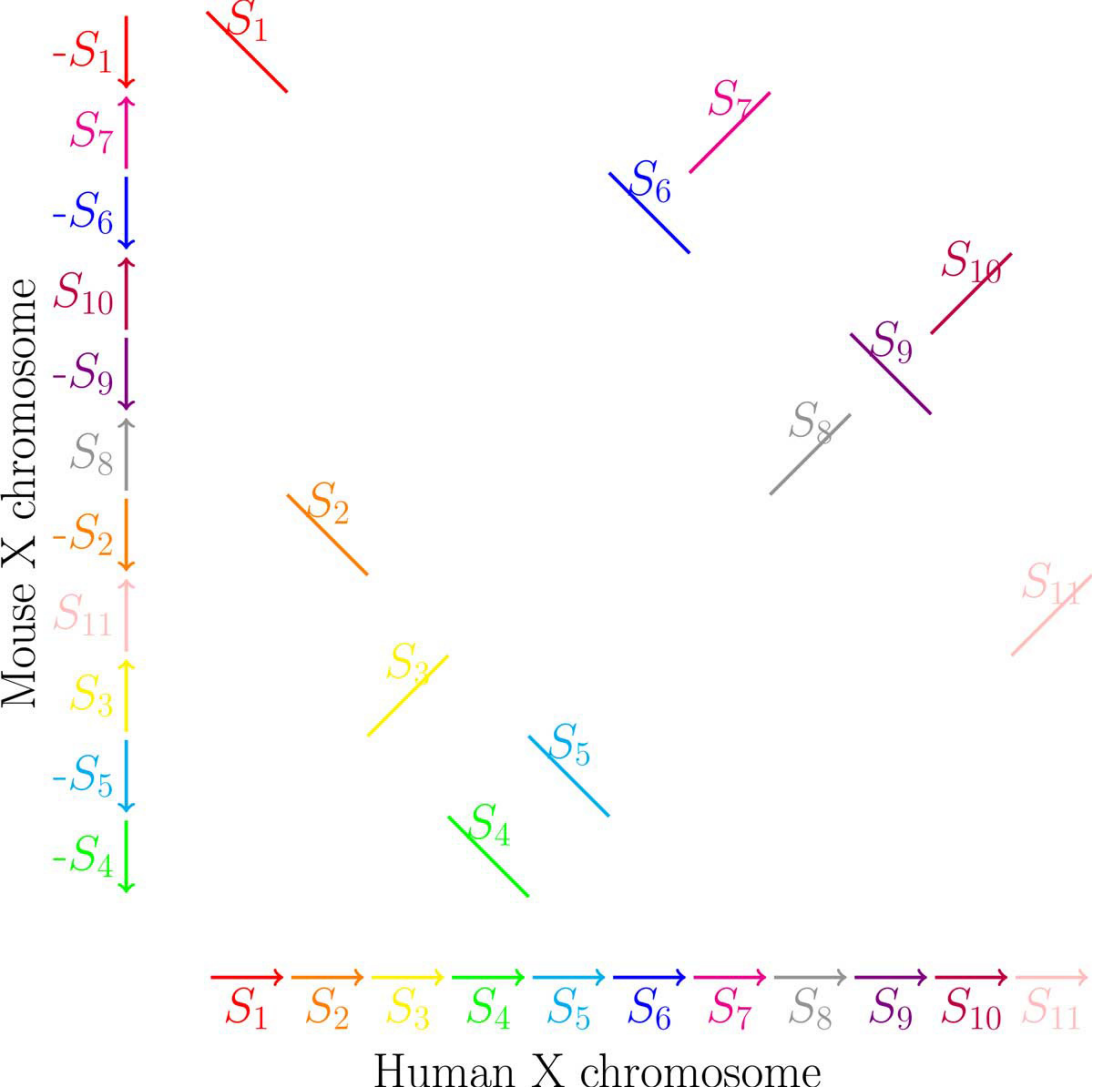
**The 11 synteny blocks shared by Human and Mouse × chromosomes (adapted from Pevzner and Tesler **[33]**)**.

**Figure 3:**
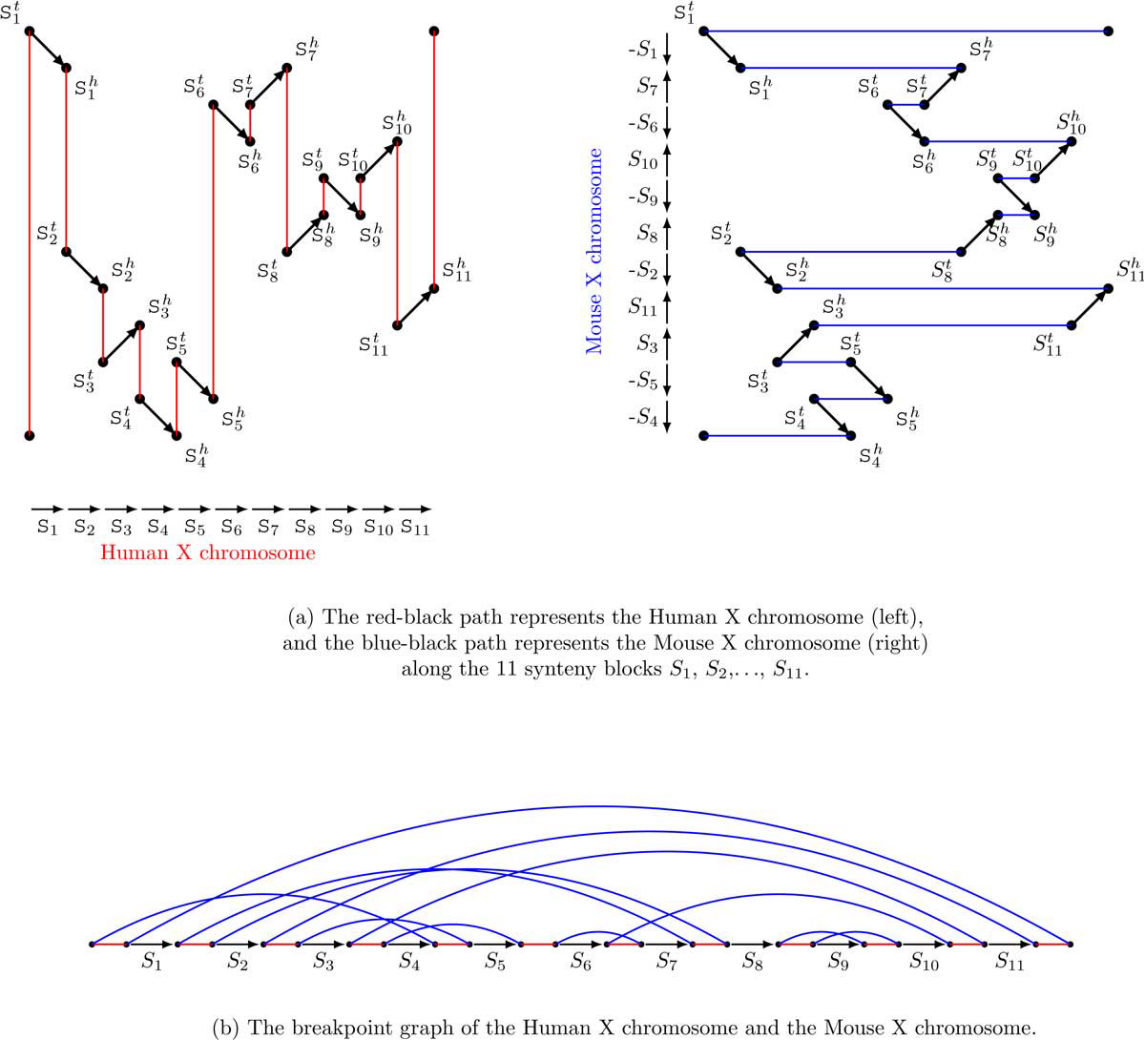
**The synteny blocks shared by Human and Mouse × chromosomes and the resulting breakpoint graph**. Human × chromosome is represented as a path formed by alternating red and black edges, while Mouse × chromosome is represented as a path formed by alternating blue and black edges.

Genome rearrangement studies usually start from constructing a set of *synteny blocks *shared by two genomes (see Figure 2). Each genome is defined as a sequence of synteny blocks separated by *breakpoint regions *and is represented as a path formed by alternating colored and black edges, where synteny blocks correspond to directed and labeled black edges and breakpoint regions correspond to undirected colored edges. Figure 3(a) presents paths corresponding to 11 synteny blocks shared by Human and Mouse × chromosomes. Each synteny block *S_i _*is represented as an directed black edge $\left(S_{i}^{t},S_{i}^{h}\right)$, where $S_{i}^{t}$ and $S_{i}^{h}$ refer to the endpoints of the synteny blocks representing its tail and head, respectively. Two consecutive synteny blocks are separated by a breakpoint region in the Human (Mouse) × chromosome that is modeled by a red (blue) edge connecting the corresponding endpoints of these synteny blocks. The (traditional) breakpoint graph of Human and Mouse × chromosomes is obtained by "gluing" identically labeled black edges in these two paths as shown in Figure 3.

The multiple breakpoint graphs [27] are constructed from a set of *k *> 2 genomes using the same procedure. While every synteny block appears just once in each of the genomes shown above, the definition of the breakpoint graph naturally extends to the case when a synteny block appears multiple times (or does not appear in a particular genome).

The classical de Bruijn graph is defined on a single genome (represented as a DNA string), while the classical breakpoint graph is defined on two genomes (represented as sequences of synteny blocks). Since synteny blocks are DNA strings, the question arises whether one can study both the de Bruijn graph and the breakpoint graph in the same framework, and what is the difference between the de Bruijn graph and the breakpoint graph.

In this paper, we generalize the definitions of both the de Bruijn graph and the breakpoint graph for both single and multiple genomes and for both single-stranded and double-stranded cases. We further show that the breakpoint graph and the de Bruijn graph are mathematically equivalent.

## A single genome (single-stranded case)

**Figure 4:**
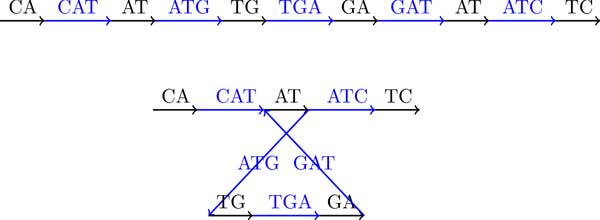
**The breakpoint graph *BP *(*CATGATC*, 3)**. 2-mers represent synteny blocks and 3-mers represent breakpoint regions.

We now generalize the definition of the breakpoint graph to a single DNA strings. Traditionally, breakpoint graphs were defined on the set of synteny blocks rather than directly on DNA strings. Given a string *String*, we define its "*synteny blocks*" as its (*k*-1)-mers, represent each (*k*-1)-mer by a directed black edge, and construct an alternating path, denoted *Path_BP _*(*String, k*), by inserting directed colored edges between consecutive (*k*-1)-mers. The breakpoint graph is obtained by gluing identical (*k*-1)-mers (directed black edges) in this path. It is easy to see that each breakpoint region connects 2 consecutive (*k*-1)-mers and thus corresponds to a kmer. The breakpoint graph of a string *String*, denoted *BP *(*String, k*), is shown in Figure 4.

**Figure 5:**
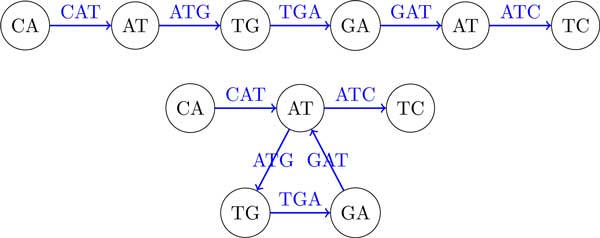
**The de Bruijn graph **DB**(*CATGATC*, 3)**. 2-mers are represented by vertices and 3-mers are represented by edges.

Given a string *String *= *s*_1_*s*_2 _. . . *s_n_*, the de Bruijn graph *DB*(*String, k*) is defined as follows. The string *String *is represented as a colored path *Path_DB _*(*String, k*), whose (*n *− *k *+ 1) edges are labeled by *k*-mers, *s*_1_*s*_2 _. . . *s_k_, s*_2_*s*_3 _. . . *s_k+1_*, . . ., and *s*_*n*−*k*+1_s_n−*k*+2 _. . . *s_n_*. Implicitly, each vertex in the path is labeled by a (*k*-1)-mer. The de Bruijn graph *DB*(*String, k*) results from gluing identically labeled vertices in the path [28]. Figure 5 shows the de Bruijn graph on the same string as in Figure 4.

Comparison of Figure 4 and 5 reveals that *BP *(*String, k*) is equivalent to *DB*(*String, k*). *BP *(*String, k*) can be converted into *DB*(*String, k*) by collapsing all black edges, while *DB*(*String, k*) can be converted into *BP *(*String, k*) by expanding each vertex v into a directed edge (*v′*, *v″*) in such a way that all incoming edges into *v *(outgoing edges from *v*) become incoming edges into *v′ *(outgoing edges from *v″*). Table 1 summarizes the correspondence between *BG*(*String, k*) and *DB*(*String, k*).

**Table 1:** The correspondence between *BP*(*String, k*) and *DB*(*String, k*).

	*DB*(*String, k*)	*BP *(*String, k*)
(*k*-1)-mer	a vertex	a black directed edge

*k*-mer	a directed blue edge	a directed blue edge

glue	the vertex	the black directed edge

synteny block	the vertex	the black directed edge

breakpoint region	the directed blue edge	the directed blue edge

**Figure 6:**
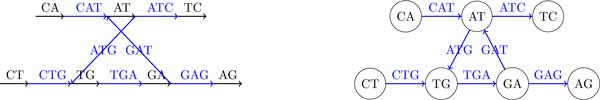
**The the breakpoint graph (left) and the de Bruijn graph (right) of two strings *CATGATC *and *CTGAG***.

We note that while the notions of the breakpoint graph and the de Bruijn graph were defined for a single string, the same definition works for a set of strings (see Figure 6).

## Multiple genomes (single-stranded case)

Given genomes *G_A _*and *G_B _*(represented as sets of strings), we classify their *k*-mers into 3 classes: *A *(occur only in *G_A_*), *B *(occur only in *G_B_*) and *AB *(occur in both *G_A _*and *G_B_*).

**Figure 7:**
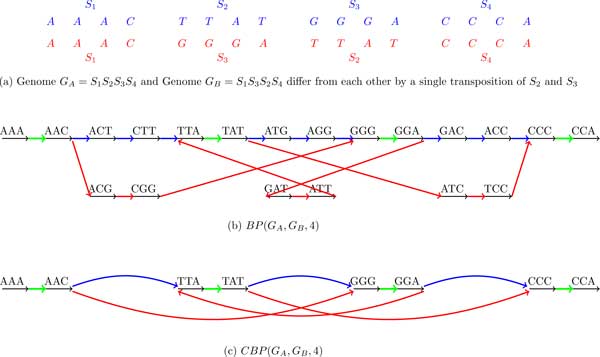
**The breakpoint graph *BP*(*G_A_, G_B_*, 4) and the condensed breakpoint graph *CBP*(*G_A_, G_B_*, 4) of two genomes *G_A _*and *G_B_***.

**Figure 8:**
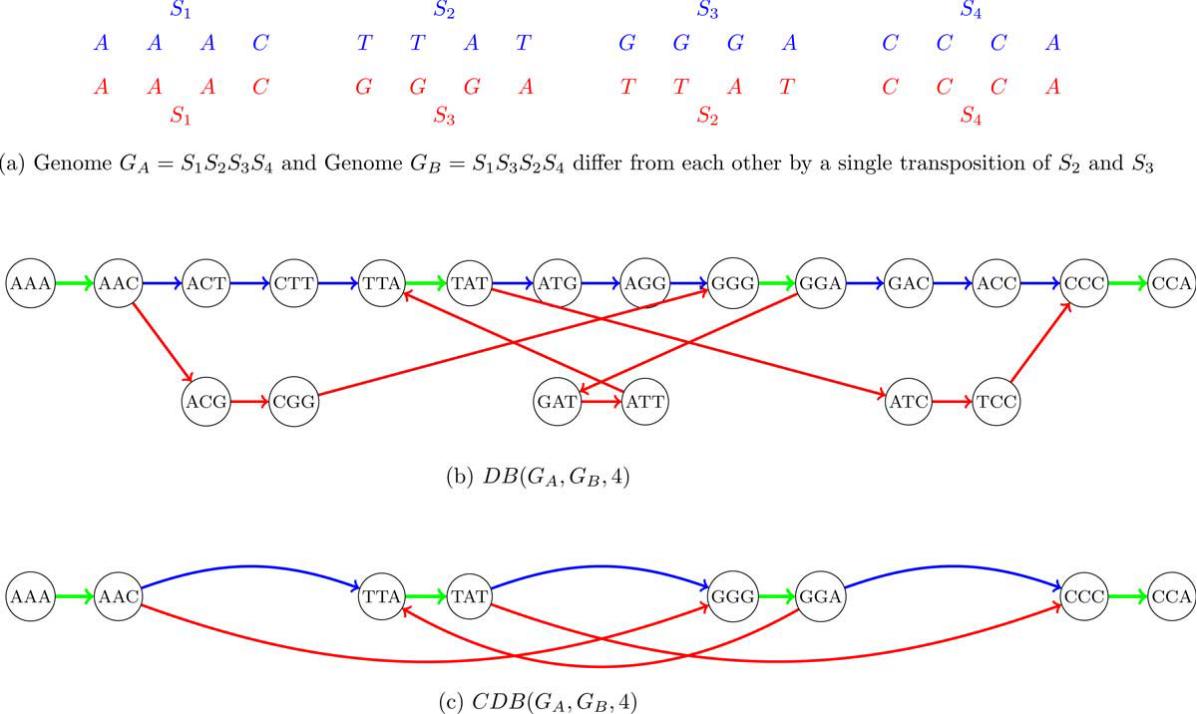
**The de Bruijn graph *DB*(*G_A_, G_B_*, 4) and the condensed de Bruijn graph *CDB*(*G_A_, G_B_*, 4) of two genomes *G_A _*and *G_B_***.

The breakpoint graph *BP *(*G_A_, G_B_, k*) is simply coloring the *k*-mer edges of the breakpoint graph *BP*(*G_A _*∪ *G_B_, k*) into 3 colors: *A *(blue), *B *(red) and *AB *(green) (Figure 7). Similarly, the de Bruijn graph *DB*(*G_A_, G_B_, k*) is simply coloring the edges of the de Bruijn graph *DB*(*G_A _*∪ *G_B_, k*) into 3 colors: *A *(blue), *B *(red) and *AB *(green) (see Figure 8).

In practice, both *BP *(*G_A_, G_B_, k*) and *DB*(*G_A_, G_B_, k*) are often *condensed *as follows.

A non-branching directed path consisting of 3 edges is called *condensible *in *BP*(*G_A_, G_B_, k*) if its middle edge is black and its starting and ending edges have the same color *C*. We substitute a condensible path by a single directed edge colored *C *with the same direction as the direction of the path. The condensed breakpoint graph *CBP*(*G_A_, G_B_, k*) iteratively replaces all condensible paths by single edges in *BP*(*G_A_, G_B_, k*) (Figure 7(c)). A new edge resulting from condensing a nonbranching path formed by edges *e*_1_, *e*_2_, and *e*_3 _is assigned a label whose prefix is a label of *e*_1 _and whose suffix is the label of *e*_3 _(labeling of edges in the condensed graphs is not the focus of this paper and is not discussed in the examples below).

A non-branching directed path consisting of 2 edges is called *condensible *in *DB*(*G_A_, G_B_, k*) if its starting and ending edges have the same color *C*. We substitute a condensible path by a single edge colored *C *with the same direction as the direction of the path. The condensed de Bruijn graph *CDB*(*G_A_, G_B_, k*) iteratively replaces all condensible paths by single edges in *DB*(*G_A_, G_B_, k*) (Figure 8(c)).

Note that both the condensed breakpoint graph in Figure 7(c) and the condensed de Bruijn graph in Figure 8(c) reveal an alternating red-blue cycle on 6 edges, a signature of a transposition. While researchers working on genome rearrangements are well aware about alternating cycles in the breakpoint graphs, the researchers working on de Bruijn graphs may not know that these cycles represent fingerprints of rearrangements.

Figure 7 and 8 illustrate that *BP*(*G_A_, G_B_, k*) is mathematically equivalent to *DB*(*G_A_, G_B_, k*). *BP *(*G_A_, G_B_, k*) can be converted into *DB*(*G_A_, G_B_, k*) by collapsing all black edges, while *DB*(*G_A_, G_B_, k*) can be converted into *BP*(*G_A_, G_B_, k*) by expanding all vertices. It is the same for the condensed case. Table 2 summarizes the comparison between *DB*(*G_A_, G_B_, k*) and *BP*(*G_A_, G_B_, k*).

**Table 2:** The correspondence between *DB*(*G_A_, G_B_, k*) and *BP*(*G_A_, G_B_, k*) (under the single-stranded representation).

	*DB*(*G_A_, G_B_, k*)	*BP*(*G_A_, G_B_, k*)
(*k*-1)-mer	vertex	black directed edge (*E*_1_)

*k*-mer	directed edge	directed edge (*E*_2_)

color	red/blue/green directed edge blue in *G_A_*, red in *G_B_*, green in both *G_A _*and *G_B_*	red/blue/green directed edge (*E*_2_) blue in *G_A_*, red in *G_B_*, green in both *G_A _*and *G_B_*

glue	vertex	*E*_1_

synteny block as a path	vertex-green edge-. . .-vertex	*E*_1 _-green *E*_2_-. . .-*E*_1_

breakpoint region as a path	red edge-vertex. . .-red edge blue edge-vertex-. . .-blue edge	red *E*_2 _-*E*_1_-. . .-red *E*_2_ blue *E*_2 _-*E*_1_-. . .-blue *E*_2_

condensing paths into edges	red edge-vertex-red edge → red edge blue edge-vertex-blue edge → blue edge green edge-vertex-green edge → green edge	red *E*_2 _-*E*_1_-red *E*_2 _→ red *E*_2 _blue *E*_2_-*E*_1_-blue *E*_2 _→ blue *E*_2_ green *E*_2_-*E*_1_-green *E*_2 _→ green *E*_2_

after condensation	*CDB*(*G_A_, G_B_, k*)	*CBP*(*G_A_, G_B_, k*)

synteny block in condensed graph	vertex-green edge-vertex	*E*_1_-green *E*_2_-*E*_1_

breakpoint region in condensed graph	red edge blue edge	red *E*_2_ blue *E*_2_

We refer to black directed edges in the de Bruijn graph as *E*_1_-edges and to colored directed edges as *E*_2_-edges

While the above notions of the breakpoint graph and the de Bruijn graph were defined for 2 genomes, they naturally generalize to any number of genomes [27].

## A single genome (double-stranded case)

We now generalize the notions of the de Bruijn and breakpoint graphs from single-stranded to double-stranded genomes. Instead of the explicit representation of both strands (like in most existing assemblers), we describe a more efficient representation that encodes both strands in a single *canonical strand *(compare with similar representations of both strands in SPAdes [12] and some other assemblers).

For a nucleotide *x*, we denote its complementary nucleotide as $\bar{x}$, e.g., $\bar{A}=T$ T and $\bar{C}=G$. Given a *k*-mer *s *= *s*_1_*s*_2 _. . . *s*_k_, we define its *reverse complement *as the *k*-mer $\bar{s}=\bar{s_{k}}\ldots \bar{s_{2}}\;\bar{s_{1}}$. We call a *k*-mer *canonical *if it is smaller or equal (in the lexicographic order) than its reverse complement. For example, AG and AT are canonical 2-mers but CT is not a canonical 2-mer. In this section, we will represent strings as paths labeled only by canonical *k*-mers and will define the breakpoint and de Bruijn graphs as the results of gluing these paths.

**Figure 9:**
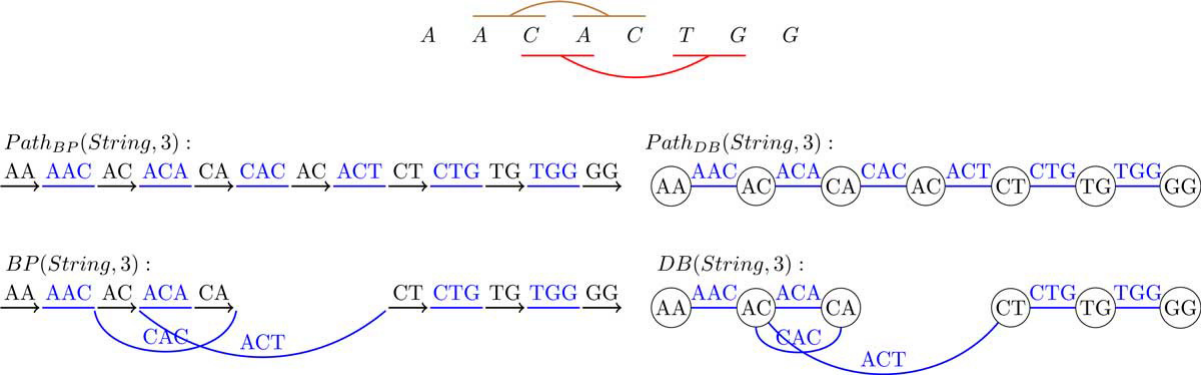
**A string *String *= *ACAGTC A *(top), *BP*(*String*, 3) (left) and *DB*(*String*, 3) (right)**. A pair of repeats in *String *is shown in brown and a pair of inverted repeats is shown in red.

While *DB*(*String, k*) and *BP *(*String, k*) glue *identical *(*k*-1)-mers in *String*, these graphs were not designed to glue a (*k*-1)-mer with its reverse complement (see Figure 9). In many applications, gluing (*k*-1)-mers with their reverse complements would be beneficial; for example, developers of many genome assemblers invest significant efforts in maintaining the "symmetric" de Bruijn graphs at all stages of the assembly so that the subgraph representing one strand is topologically identical to the subgraph representing another strand [12]. Since the breakpoint graph and the de Bruijn graph in Figure 9 do not allow one to analyze the interplay between rearrangements and inverted repeats, we need to come up with a new graph-theoretical model for double-stranded genomes. We note that since A-Bruijn graphs were designed to accommodate gluing of *arbitrary *positions in *String *(as long as they are defined as "aligned" in the A-Bruijn graph framework [13]), gluing (*k*-1)-mers with their reverse complements perfectly fits in the framework of the A-Bruijn graphs.

**Figure 10:**
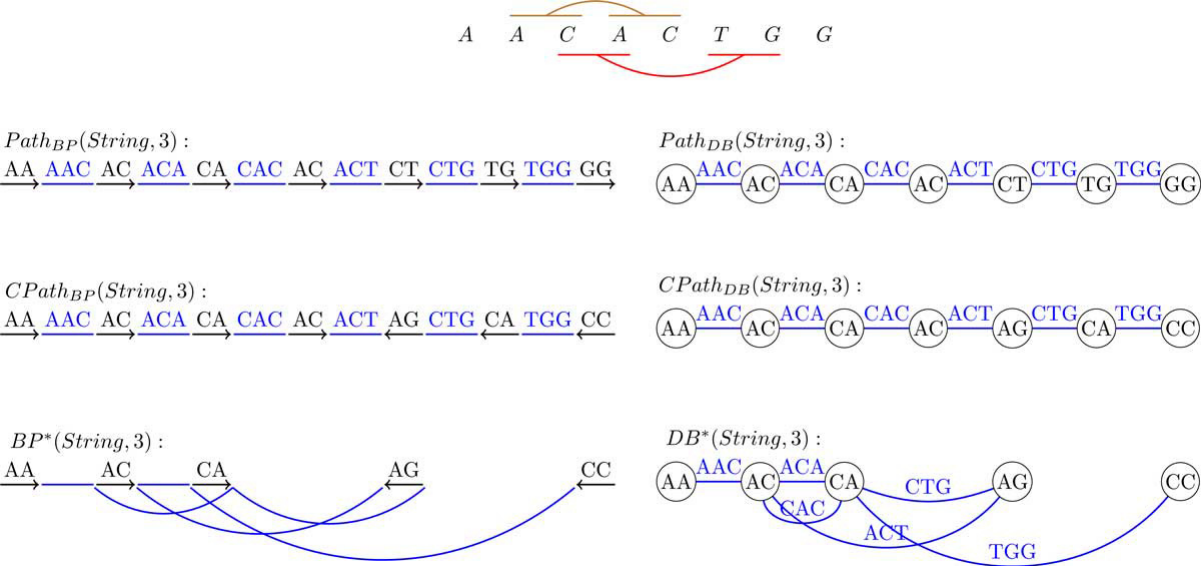
A string *String *= *ACAGTCA *(top) represented by canonical paths *CPath_BP _*(*String*, 3) and *CPath_DB _*(*String*, 3), the double-stranded breakpoint graph BP*(*String*, 3) (left) and the double-stranded de Bruijn graph DB*(*String*, 3) (right). A pair of repeats in *String *is shown in brown and a pair of inverted repeats is shown in red.

To model double-stranded strings as paths, we introduce the concept of a *canonical *path representing a string *String *(that differs from the standard representation of *String *as a path from Section 2). To transform a standard path *Path_BP _*(*String, k*) into a canonical path *CPathBP *(*String, k*), we reverse directions of all black edges labeled by non-canonical (*k*-1)-mers and change their labels into their reverse complements (see Figure 10, left). As a result, all black edges in the canonical path are labeled by canonical (*k*-1)-mers.

To transform a standard path *Path_DB _*(*String, k*) into a canonical path *CPath_DB _*(*String, k*), we simply change labels of all vertices labeled by non-canonical (*k*-1)-mers into their reverse complements (see Figure 10, right).

After transforming standard paths *Path_BP _*(*String, k*) and *Path_DB _*(*String, k*) into a canonical paths *CPath_BP _*(*String, k*) and *CPath_DB _*(*String, k*) respectively, the definition of the breakpoint graph (gluing of identically labeled black edges in *CPath_BP _*(*String, k*)) and the de Bruijn graph (gluing of identically labeled vertices in *CPath_BP _*(*String, k*)) remain unchanged. Pairs of *k*-mer edges are also glued if they represent the reverse complement *k*-mer of each other and are labeled by both *k*-mers. The graphs obtained after gluing these paths are called the double-stranded breakpoint graph BP* (*String, k*) and the double-stranded de Bruijn graph DB* (*String, k*) (Figure 10). While BP*(*String, k*) makes use of the direction of the black edge to represent whether the canonical string or its reverse complement is

used, DB* (*String, k*) collapses all black edges into vertices and no longer maintains the direction information to distinguish these two possibilities. A pair of vertices in DB* (*String, k*) labeled by canonical (*k*-1)-mers *v *and *w *may potentially correspond to 4 types of edges depending on whether the edge connects (i) *v *and *w*, (ii) *v *and $\bar{w}$, (iii) $\bar{v}$ and *w*, and (iv) $\bar{v}$ and $\bar{w}$ (compare to the *bi-directed de Bruijn graph *[29]).

As before, DB*(*String, k*) is obtained from BP*(*String, k*) by collapsing all black edges, while BP*(*String, k*) is obtained from DB*(*String, k*) by expanding all vertices into black edges (and connecting black edges according to the labels on the colored edges).

One may notice that while the double-stranded de Bruijn graph is similar to the bi-directional de Bruijn graph introduced in [29], it does not require the explicit introduction of the bi-directional edges. The notions of the double-stranded breakpoint graphs and de Bruijn graphs also can be naturally extended from a single double-stranded string to multiple double-stranded strings.

## Multiple genomes (double-stranded case)

Given a string *String *= *s*_1_*s*_2 _. . . *s*_i−1_*s_i_s_i+1 _*. . . *s_j−1 _s_j _s_j+1 _*. . . *s_n−1_s_n_*, a reversal of the segment *s_i_s_i+1 _*. . . *s_j−1_*s_j _results in a string $s_{1}s_{2}\cdots \bar{s_{j}}\;\bar{s_{j-1}}\cdots \bar{s_{i+1}}\;\bar{s_{i}}\;s_{j+1}\cdots s_{n-1}s_{n}$ where this segment is substituted by its reverse complement. Below we illustrate that reversals are represented as usual alternating blue-red cycles in the condensed double-stranded breakpoint and de Bruijn graphs.

Given two genomes *G_A _*and *G_B_*, we classify each *k*-mer s into 3 classes: *A *(if either *s *or $\bar{s}$ belongs to *G_A _*but neither *s *or $\bar{s}$ belongs to *G_B_*), *B *(if either *s *or $\bar{s}$ belongs to *G_B _*but neither *s *or $\bar{s}$ belongs to *G_A_*) and *AB *(if both *G_A _*and *G_B _*contain either *S *or $\bar{S}$).

**Figure 11:**
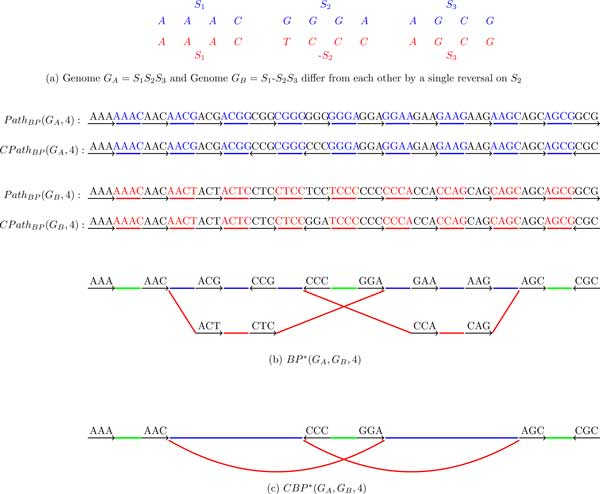
The double-stranded breakpoint graph BP*(*G_A_, G_B_*, 4) and the condensed double-stranded breakpoint graph CBP*(*G_A_, G_B_*, 4) of two genomes *G_A _*and *G_B_*. Each of 3 synteny blocks in both BP*(*G_A_, G_B_*, 4) and CBP* (*G_A_, G_B_*, 4) is represented as a path (starting and ending in black edges and having a green edge in the middle).

**Figure 12:**
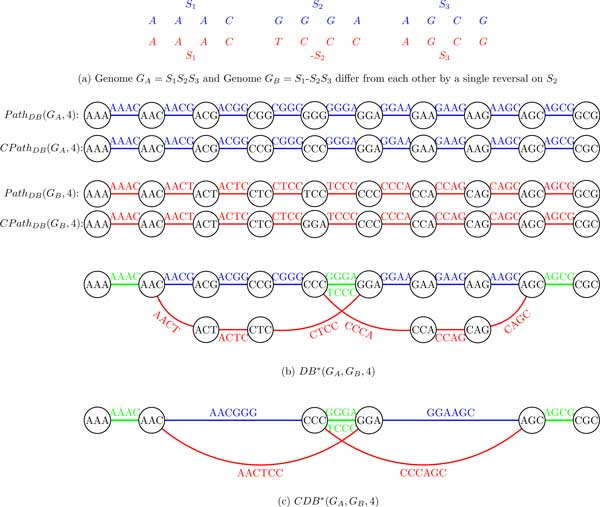
The double-stranded de Bruijn graph DB*(*G_A_, G_B_*, 4) and the condensed double-stranded de Bruijn graph CDB*(*G_A_, G_B_*, 4) of genomes *G_A _*and *G_B_*.

The double-stranded breakpoint graph BP*(*G_A_, G_B_, k*) is simply coloring the edges in BP* (*G_A _*∪ *G_B_, k*) into 3 colors: *A *(blue), *B *(red) and *AB *(green) (Figure 11). Similarly, the double-stranded de Bruijn graph DB*(*G_A_, G_B_, k*) is simply coloring the edges in DB*(*G_A _*∪ *G_B_, k*) into 3 colors: *A *(blue), *B *(red) and *AB *(green) (Figure 12).

Both BP*(*G_A_, G_B_, k*) and DB*(*G_A_, G_B_, k*) can be further condensed as described in Table 2 resulting in the condensed double-stranded breakpoint graph CBP* (*G_A_, G_B_, k*) and the condensed double-stranded de Bruijn graph CDB*(*G_A_, G_B_, k*), respectively (Figure 11(c) and Figure 12(c)).

As before, BP*(*G_A_, G_B_, k*) (CBP*(*G_A_, G_B_, k*)) is obtained from DB*(*G_A_, G_B_, k*) (CDB*(*G_A_, G_B_, k*)) by collapsing all black edges, while DB*(*G_A_, G_B_, k*) (CDB* (*G_A_, G_B_, k*)) is obtained from BP*(*G_A_, G_B_, k*) (CBP*(*G_A_, G_B_, k*)) by expanding all vertices into black edges (and connecting black edges according to the labels on the colored edges).

While the above notions of the double-stranded breakpoint graph and the double-stranded de Bruijn graph were defined for 2 genomes, they naturally generalize to any number of genomes.

## Conclusion

We described the connection between the breakpoint graph and the de Bruijn graph that reveals that these constructions (that have been treated as two different data structures for over two decades) are essentially identical. We believe that the explicit description of this connection will contribute to a dialog between two previously separated bioinformatics communities studying genome rearrangements and genome assembly. It may also clarify the connection between the breakpoint graph, the de Bruijn graph, and the string graph introduced by Myers [30], another powerful paradigm for genome assembly. As hinted by Pop [31], the string graphs are functionally equivalent to the de Bruijn graphs, e.g., the comparison of Figure 1 in [30] and Figure 2 in [13]) suggests that the string graph is a special case of the ABruijn graph. Simpson and Durbin [32] further suggested that the de Bruijn graph and the string graph constructions on all the k-mers of a genome (using parameter *τ *= *k *− 1 for the string graph) are equivalent. However, the explicit description of this equivalence is still missing and we hope that the proposed A-Bruijn graph framework can be further extended to cover the string graphs as well.

## Competing interests

The authors declare that they have no competing interests.

## Authors' contributions

PAP conceived the study. YL, SN and PAP performed the analysis. YL and PAP wrote the paper with help from SN. All authors read and approved the final manuscript.

## Declarations

Publication costs for this article were funded by St. Petersburg Academic University.

This article has been published as part of *BMC Genomics *Volume 15 Supplement 6, 2014: Proceedings of the Twelfth Annual Research in Computational Molecular Biology (RECOMB) Satellite Workshop on Comparative Genomics. The full contents of the supplement are available online at http://www.biomedcentral.com/bmcgenomics/supplements/15/S6.

## References

[B1] Pevzner PA l-tuple DNA sequencing: computer analysis J Biomol Struct Dyn 1989 7 63 73 2684223 10.1080/07391102.1989.10507752

[B2] Idury RM Waterman MS A new algorithm for DNA sequence assembly J Comput Biol 1995 2 2 291 306 10.1089/cmb.1995.2.291 7497130

[B3] Pevzner PA Tang H Waterman MS An Eulerian path approach to DNA fragment assembly Proc Nat'l Acad Sci USA 2001 98 17 9748 10.1073/pnas.171285098 11504945 PMC55524

[B4] Zerbino DR Birney E Velvet: algorithms for de novo short read assembly using de Bruijn graphs Genome Research 2008 18 5 821 829 10.1101/gr.074492.107 18349386 PMC2336801

[B5] Chaisson MJ Pevzner PA Short read fragment assembly of bacterial genomes Genome Research 2008 18 2 324 330 10.1101/gr.7088808 18083777 PMC2203630

[B6] Simpson JT Wong K Jackman SD Schein JE Jones SJM Birol I Abyss: a parallel assembler for short read sequence data Genome Research 2009 19 6 1117 1123 10.1101/gr.089532.108 19251739 PMC2694472

[B7] Peng Y Leung H Yiu S Chin F IDBA - a practical iterative de Bruijn graph de novo assembler Proc 14th Int'l Conf Comput Mol Biol (RECOMB'10) Lecture Notes in Comp Sci 2010 6044 426 440

[B8] Butler J MacCallum I Kleber M ALLPATHS: de novo assembly of whole-genome shotgun microreads Genome Research 2008 18 5 810 820 10.1101/gr.7337908 18340039 PMC2336810

[B9] Boisvert S Laviolette F Corbeil J Ray: simultaneous assembly of reads from a mix of high-throughput sequencing technologies J Comput Biol 2010 17 11 1519 1533 10.1089/cmb.2009.0238 20958248 PMC3119603

[B10] Li R Zhu H Ruan J De novo assembly of human genomes with massively parallel short read sequencing Genome Research 2010 20 2 265 272 10.1101/gr.097261.109 20019144 PMC2813482

[B11] Chitsaz H Yee-Greenbaum JL Tesler G Efficient de novo assembly of single-cell bacterial genomes from short-read data sets Nature biotechnology 2011 21926975 10.1038/nbt.1966 PMC3558281

[B12] Bankevich A Nurk S SPAdes: A new genome assembly algorithm and its applications to single-cell sequencing J Comput Biol 2012 19 5 455 477 10.1089/cmb.2012.0021 22506599 PMC3342519

[B13] Pevzner PA Tang H Tesler G De novo repeat classification and fragment assembly Genome Research 2004 14 9 1786 1796 10.1101/gr.2395204 15342561 PMC515325

[B14] Böcker S Sequencing from compomers: Using mass spectrometry for dna de novo sequencing of 200+ nt J Comput Biol 2004 11 6 1110 1134 10.1089/cmb.2004.11.1110 15662201

[B15] Pham SK Pevzner PA DRIMM-Synteny: decomposing genomes into evolutionary conserved segments Bioinformatics 2010 26 20 2509 2516 10.1093/bioinformatics/btq465 20736338

[B16] Minkin I Patel A Kolmogorov M Vyahhi N Pham S Sibelia: a scalable and comprehensive synteny block generation tool for closely related microbial genomes Proc 13th Workshop Algs in Bioinf (WABI'13) Lecture Notes in Comp Sci 2013 8126 215 229 10.1007/978-3-642-40453-5_17

[B17] Raphael B Zhi D Tang H Pevzner PA A novel method for multiple alignment of sequences with repeated and shuffled elements Genome Research 2004 14 11 2336 2346 10.1101/gr.2657504 15520295 PMC525693

[B18] Bafna V Pevzner PA Genome rearrangements and sorting by reversals Proc 34th Ann IEEE Symp Foundations of Comput Sci (FOCS'93) 1993 148 157

[B19] Fertin G Labarre A Rusu I Tannier E Vialette S Combinatorics of Genome Rearrangements MIT Press, Inc

[B20] Iqbal Z Caccamo M Turner I Flicek P McVean G De novo assembly and genotyping of variants using colored de bruijn graphs Nature genetics 2012 44 2 226 232 10.1038/ng.1028 22231483 PMC3272472

[B21] Boussau B Daubin V Genomes as documents of evolutionary history Trends in ecology & evolution 2010 25 4 224 232 10.1016/j.tree.2009.09.007 19880211

[B22] Bailey JA Baertsch R Kent WJ Haussler D Eichler EE Hotspots of mammalian chromosomal evolution Genome Biology 2004 5 4 23 10.1186/gb-2004-5-4-r23 PMC395782 15059256

[B23] Zhao H Bourque G Recovering genome rearrangements in the mammalian phylogeny Genome Research 2009 19 5 934 942 10.1101/gr.086009.108 19411607 PMC2675982

[B24] Alekseyev MA Pevzner PA Comparative genomics reveals birth and death of fragile regions in mammalian evolution Genome Biology 2010 11 11 117 10.1186/gb-2010-11-11-r117 21118492 PMC3156956

[B25] Nurk S Pevzner PA Sparcle: using colored de bruijn graphs for analysing genome variations, unpublished manuscript

[B26] Guzman GI Utrilla J Monk JM Brunk E Ebrahim A Nurk S Palsson BO Feist AM Model-driven discovery of 'underground' isozyme functions in escherichia coli, unpublished manuscript 10.1073/pnas.1414218112 PMC4311852 25564669

[B27] Alekseyev MA Pevzner PA Breakpoint graphs and ancestral genome reconstructions Genome Research 2009 19 5 943 957 10.1101/gr.082784.108 19218533 PMC2675983

[B28] Compeau PEC Pevzner PA Bioinformatics Algorithms: An Active-Learning Approach

[B29] Medvedev P Georgiou K Myers G Brudno M Computability of models for sequence assembly Proc 7th Workshop Algs in Bioinf (WABI'07) Lecture Notes in Comp Sci 2007 4645 289 301 10.1007/978-3-540-74126-8_27

[B30] Myers EW The fragment assembly string graph Bioinformatics 2005 21 suppl 2 79 85 16204131 10.1093/bioinformatics/bti1114

[B31] Pop M Genome assembly reborn: recent computational challenges Briefings in bioinformatics 2009 10 4 354 366 10.1093/bib/bbp026 19482960 PMC2691937

[B32] Simpson JT Durbin R Efficient construction of an assembly string graph using the fm-index Bioinformatics 2010 26 12 367 373 10.1093/bioinformatics/btq217 PMC2881401 20529929

[B33] Pevzner PA Tesler G Genome rearrangements in mammalian evolution: lessons from human and mouse genomes Genome Research 2003 13 1 37 45 10.1101/gr.757503 12529304 PMC430962

